# How Is Knowledge Perceived as Power? A Multilevel Model of Knowledge Power in Innovation Networks

**DOI:** 10.3389/fpsyg.2021.630762

**Published:** 2021-10-20

**Authors:** Chengqi Shi, Fan Zhang, Peiyao Zhu, Qinlu Shi

**Affiliations:** Shaanxi University of Science and Technology, Xi’an, China

**Keywords:** knowledge power, inter-organizational power relation, knowledge-based view, innovation network, activated knowledge, power perception

## Abstract

Inter-organizational power relations have long been considered to be balanced in innovation networks, which are viewed as loosely coupled systems. Some recent studies, however, show that innovation networks are asymmetric and hierarchical, and the power of network actors has become a significant but rarely addressed issue. As knowledge is the most important resource in the network, this paper introduces the concept of knowledge power by combining related research perspectives and conducting some fundamental research on it as follows: (1) knowledge power’s origins are analyzed by proposing the term “activated knowledge” and studying the path through which it is formed over multiple levels of the network; (2) a multilevel framework of characteristics of activated knowledge, which is considered the major determinant of knowledge power, is established, and suggestions are offered for how they impact knowledge power; and (3) a multilevel measurement model for knowledge power is built, and the above propositions are tested by mathematical inference. The purpose of this paper is not only to study knowledge power’s formation, determinants, and measurement but also to offer a comprehensive view, combining multiple network levels and multiple research perspectives, that should be useful to researchers conducting future studies in this field.

## Introduction

Networks are composed of inter-organizational relations. At present, the knowledge-based view (KBV) is an important framework for the study of inter-organizational relations (Ireland et al., 2002; Jha and Cottam, 2021) and continues to attract attention (Heiman and Nickerson, 2004; Quintane et al., 2011; Jordão, 2015; Lyu et al., 2020). In the KBV, network actors are viewed as knowledge sets in an innovation network, and the research focus is on individual organizations’ knowledge structures and intra- or inter-organizational knowledge transfers (Kim, 2015; Ouakouak et al., 2021). This trend, however, gives rise to the following problems: first, the current studies rarely combine resource dependencies based on the KBV with power mechanisms, although the interdependence of knowledge leads to power (Casciaro and Piskorski, 2005; Marjolein and Caniëls, 2009; Back and Kohtamäki, 2015) and power can be seen as a mechanism for achieving coordination and cooperation among network members (Oliver, 1991; Dhanaraj and Parkhe, 2006; Matheus et al., 2017). Second, innovation networks have multiple levels (Contractor et al., 2006; Provan et al., 2007; Matinheikki et al., 2017). The KBV mainly focuses on organizational knowledge characteristics and intra- or inter-organizational knowledge transfers (Lai et al., 2016) but neglects knowledge distribution over the whole network. Third, innovation networks are usually abstracted as knowledge networks (Samarra and Biggiero, 2008; Dantas and Bell, 2009) based on the KBV. This leads to research in this area that tends to emphasize organizational knowledge rather than individual organizations in the network and to ignore organizational strategic aims (Gilsing et al., 2007; An et al., 2021) and the role of network members (Na et al., 2020).

It is not enough to study innovation networks from the perspective of the KBV alone because knowledge and capabilities are both key elements of an enterprise (Santoro et al., 2021). Enterprises have many capabilities, among which dynamic capabilities are particularly important. The term “dynamic capability” (DC) refers to the renewal of resources and competencies to address changing environments (Eisenhardt and Martin, 2000). This term is, thus, closely related to the KBV and can also extend it, as many researchers find that DCs can have active impacts on enterprises in an innovation context. For example, DCs can influence firm performance through a variety of means and mechanisms (Torres et al., 2018; Prester et al., 2019; Ferreira et al., 2020). There are positive correlations between DCs and marketing capability, operation capability, and new product development performance (Mu, 2017); DCs can have a significant positive effect on short-term financial performance and long-term competitive advantage (Liu et al., 2019). In addition, existing research has also explored the application of DCs in different environments, such as the highly dynamic background of digital strategy (Yeow et al., 2018) and underlying organizational routines (Mousavi et al., 2018).

DCs can reflect an enterprise’s agency, but both knowledge and capability are limited as features of an individual organization. As a special networked form of organization, the innovation network falls somewhere between a market and a hierarchy and is often viewed as a collection of loosely coupled systems belonging to autonomous firms (Orton and Weick, 1990; Dhanaraj and Parkhe, 2006; Papadonikolaki, 2018; Su et al., 2021). Initiatives by members and interactions between members should be most emphasized. Studies in this field are generally based on the assumption that inter-organizational power relations are balanced and that there is equality between network actors. Some recent studies, however, show that innovation networks are asymmetric (Cowan and Jonard, 2009; Hao and Feng, 2018), with a pronounced hierarchical structure being observed (Powell et al., 2005; Brenner et al., 2011). Especially with the rapid pace of competition and the constant updates to technology, the formation and running of an innovation network is increasingly a result of conscious and organized behaviors of organizations focused on a common technology-innovation task (Podolny and Page, 1998; Cowan et al., 2007; Yang, 2020). Some of the latest studies show that power is very important or even dominant in R&D collaborations and networks (Back and Kohtamäki, 2015; Bujor and Avasilcăi, 2018; Hao and Feng, 2018; Papadonikolaki, 2018; Valdez, 2018). There are strong relationships among attraction, dependency, and power (Hald et al., 2009; Ramsay and Wagner, 2009; Jakobsen et al., 2019). Therefore, power can provide a new and unique perspective for solving the above research problems. In this sense, this paper introduces the term “knowledge power” into the study of technology-innovation networks and defines it as the inter-organizational power-dependence relation that is formed on the basis of organizational knowledge and is eventually manifested by organizational positions in a “power network.”^1^ It thus matches the multilevel feature of the innovation network and combines multiple research perspectives. Also, network actors’ active roles in the network are revealed well by the exertion of their power and the power interactions among them. However, questions such as how knowledge power comes into being, what factors shape it, and how to measure it remain challenging and unsolved. Therefore, this paper aims to (1) illustrate knowledge power’s formation path in innovation networks by synthesizing the relevant literature; (2) offer suggestions for how organizational knowledge characteristics impact it at different network levels; and (3) provide a multilevel measurement model for it, and test the propositions by mathematical inference.

## Formation of Knowledge Power

### Origin

In sociology, Foucault and Gordon (1980) takes knowledge as being always inextricably enmeshed in relations of power in that the base of power is knowledge and the use of power is to apply knowledge. Latiff and Hassan (2008) advance the term “knowledge power,” which is derived from the control of knowledge. In innovation networks, the origin of power from knowledge is most obvious. The aim of organizations in entering into technology-innovation alliances is to profit from the knowledge possessed by others (Cassiman and Veugelers, 2006; Jorge et al., 2021). They pool their knowledge and use it as an input into new knowledge production, and repeated alliance formation creates a network (Cowan et al., 2007). An organization’s knowledge characteristics, therefore, determine its attractiveness as a knowledge supplier in the network (Pérez-Nordtvedt et al., 2008; Pulles et al., 2014). Its bargaining power and indispensability are positively associated with its ability to retain control rights to intellectual assets (Leiponen, 2008), and differences in organizations’ knowledge characteristics predict the degree of power one unit has over another (Wong et al., 2008).

Power is not derived exclusively from knowledge. Power has its roots not only in the knowledge asymmetry among organizations but also in their differences in terms of capability (Conner, 1991; Enkel et al., 2017). The term “power” is derived from the Latin word *potestas* or *potentia*, meaning capability. Clegg (1989) states that power is a capability premised on resource control. In an innovation network, an organization’s technology-innovation capability is the most fundamental and important factor determining its influence (Li et al., 2020), and the most influential organization always has stronger capabilities for searching for and absorbing useful knowledge than others (Cohen and Levinthal, 1990; Prajogo et al., 2020).

The two concepts of capability and knowledge are often interconnected. On the one hand, organizational capabilities are seen as collective knowledge (Prahalad and Hamel, 1990) or as a property of knowledge (SubbaNarasimha, 2001). On the other hand, knowledge, as a resource, is thought to be one of the most important firm capabilities (Teece et al., 1997), and the level of knowledge of a firm describes its capacity to generate technological innovation (Wersching, 2007). Several terms related to both concepts appear frequently in the research literature, such as knowledge capabilities (Dawson, 2000; Ogulin et al., 2020), knowledge-based resource capabilities (Carrillo and Gaimon, 2004), knowledge activation (Tortoriello, 2008), knowledge integration capability (Xi et al., 2020), and dynamics of capability search and creation (Helfat, 2018). These indicate that knowledge and capability can embrace each other and coexist side by side and even within each other.

In an innovation network, organizational knowledge and capabilities should not be studied separately. Knowledge is the basis for capabilities (Grant, 1995). The more profound an organization’s knowledge, the stronger its capabilities. However, knowledge is an internal and relatively static resource. It can only be applied and used through organizational capabilities and then sensed and identified by outsiders. Knowledge power derives ultimately from organizational knowledge, which, however, may not be entirely or continuously in an active state. There is “sleeping knowledge” (Charue-Duboc et al., 2010), which is knowledge that is not being used effectively or of which the organization may even be unaware. Only after being activated by capabilities can an organization’s knowledge be applied and used in technology-innovation activities, embodied through technology-innovation processes and outcomes, and sensed and identified by other network actors. Those who need but do not have the knowledge will consequently be attracted to and develop a knowledge dependence on the organization (Howard et al., 2016). A positive net dependence generates knowledge power. Therefore, this paper proposes the term “activated knowledge” and defines it as knowledge that is not only owned by an organization but also activated by its capabilities so that it is ready to be used and identified. Activated knowledge is the direct origin of knowledge power.

### Formation Path

An innovation network is a complicated form of network organization, and multilevel analyses are usually adopted in the study of such networks. A popular approach is to divide the network into three levels: the organization/firm/actor level, the inter-organizational level/dyad level/dyadic level, and the network level (Contractor et al., 2006; Provan et al., 2007). This approach considers individual organizations as the fundamental component of the network. Their micro-changes and interactions, such as cooperation and pairings, eventually result in the static structure and dynamic evolution of the macro network (Kong et al., 2019). Accordingly, this paper follows this approach and terms the three levels as the actor level, dyadic level, and network level.

Knowledge power is also multilevel in nature. Burt (1977) points out that when studying power, three general aspects must be distinguished: the bases of power (possession of resources), which are converted into manifestations of power (structure in influence relationships among decision-makers) *via* the processes of power. According to related studies (Emerson, 1962; Casciaro and Piskorski, 2005; Cho, 2020), power is a dependence relation, which in innovation networks means interdependence on one another’s knowledge. This interdependence is rooted in organizations’ heterogeneous capabilities and knowledge at the actor level and is eventually manifested as a knowledge-power network at the network level through interactions among inter-organizational relations at the dyadic level. Thus, by conducting a multilevel analysis, knowledge power can be better described as follows:

1. At the actor level: one actor alone cannot generate knowledge power, which is formed in a relational context, but organizational activated knowledge is knowledge power’s direct origin. An organization with activated knowledge favorable to the technology-innovation task will be highly attractive to other network actors. Its knowledge attractiveness leads to knowledge dependencies on the part of those who need its knowledge to accomplish the task, eventually giving rise to its knowledge power over them. Thus, at this level, knowledge power is conceptualized as knowledge attractiveness.
2. At the dyadic level: two actors’ mutual knowledge attractiveness forms an inter-organizational knowledge dependence relation. The differences in their activated knowledge result in a knowledge-dependence asymmetry that induces relative knowledge power (RKP). The party that has less unilateral knowledge dependence—and, consequently, positive RKP—is in a position of power advantage, whereas the other party is in a disadvantageous power position.
3. At the network level: multiple dyadic knowledge-power relations form a knowledge-power network, and an organization’s RKPs from various dyadic relations accumulate into a total knowledge power that is referred to as network knowledge power (NKP) in this paper. The magnitude of an organization’s NKP determines its position in the power network. Relative to most other network actors, those organizations that have bigger NKPs will occupy the central positions, and the one with the highest centrality will become the core organization, with a significant degree of influence over the whole network.

**Figure 1:**
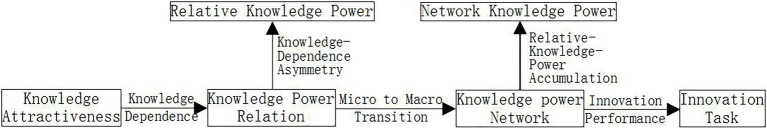
The formation path of knowledge power.

The formation path of knowledge power is shown in Figure 1.

## The Determinants of Knowledge Power

Activated knowledge is the direct origin of knowledge power, so an organization’s activated knowledge characteristics (AKCs) determine whether its knowledge power, i.e., its knowledge attractiveness, is large or small; whether its position in a dyadic relation is power advantageous or power disadvantageous; and whether it is at the core or on the periphery of the power network. This section will analyze what these characteristics are and how they impact knowledge power.

### Activated Knowledge Characteristics

Studies of the characteristics of knowledge propose various attributes such as knowledge depth, knowledge breadth, and knowledge similarity (Prabhu et al., 2005); knowledge value, knowledge rarity, knowledge inimitability, and knowledge non-substitutability (Pérez-Nordtvedt et al., 2008); and knowledge criticality, knowledge non-substitutability, and knowledge centricity (Wong et al., 2008). These studies provide the most popular and accepted characteristics of knowledge, but they neglect the possible interactions among these characteristics and do not take the multilevel nature of networks into consideration. This paper holds that a multilevel framework for AKCs should be built to perform a better investigation of how knowledge determines power in innovation networks.

1. At the actor level: AKCs should reflect the absolute level of an organization’s activated knowledge, with the purpose of measuring their impact on its knowledge attractiveness. In previous studies, factors such as knowledge depth and knowledge breadth are commonly used. Knowledge depth refers to the amount of within-field knowledge possessed by the organization, whereas knowledge breadth is the range of fields over which the organization has knowledge (Prabhu et al., 2005). These are the most fundamental knowledge characteristics and are used in this paper to measure the absolute level of an organization’s activated knowledge in its own context.
2. At the dyadic level: AKCs should reflect the relative level of an organization’s activated knowledge compared with another party, with the purpose of measuring how they determine its RKP. In previous studies, factors such as knowledge criticality, knowledge similarity, knowledge complementarity, and knowledge substitutability were commonly used. The first three all focus on the focal dyadic relation and are highly related. Knowledge similarity is in inverse proportion to knowledge complementarity within the knowledge portfolio required by the technology-innovation task. As network actors need heterogeneous knowledge or capabilities to cooperate (Pfeffer and Gerald, 1978; Wersching, 2007; Samarra and Biggiero, 2008), the less complementary (and the more similar) the focal organization’s knowledge is to that of the others, the lower the other perceives its knowledge criticality to be (Wong et al., 2008). Knowledge substitutability, however, involves the dyadic relations of the focal actor with third-network actors and measures the degree to which its knowledge can be replaced by them. It reflects the impact of other dyadic relations on the focal relation. In all, AKCs at the dyadic level are chosen as follows: knowledge complementarity that shows the degree to which one organization’s activated knowledge is complementary to the other party, and knowledge substitutability that reveals the degree to which one organization’s activated knowledge can be replaced by third parties.
3. At the network level: AKCs should reflect the relative level of an organization’s activated knowledge in the whole network, with the purpose of measuring how they determine its NKP. In previous studies, factors such as knowledge rarity, knowledge uniqueness, and knowledge centricity are commonly used. Knowledge rarity and knowledge uniqueness both show the relative level of an organization’s activated knowledge compared with the network’s average knowledge level. They are highly interrelated, as an organization’s knowledge should be unique and cannot be easily simulated by other network actors if it manages to keep its knowledge rare in the network. Also, when an organization’s knowledge is unique and difficult to copy, its knowledge rarity is usually high. Knowledge centrality shows the importance of an organization’s knowledge to the technology-innovation task. As an innovation network comes into being to accomplish the task, the more an organization’s activated knowledge meets the task’s requirements, the more important and central it is to the whole network. Therefore, AKCs at the network level are chosen as follows: knowledge rarity, i.e., the degree to which an organization’s knowledge is rare in the network, and knowledge centrality, i.e., the degree to which an organization’s knowledge is important for the technology-innovation task.

### Concept Model

1. At the actor level: the concept of attraction comes from social psychology, where it is seen as a way of bringing parties together in a voluntary manner (Hare et al., 1959; Blau, 1964). Attraction is seen as having the potential to explain why business relationships commence and develop, which is relevant to dyadic business relationships (Mortensen, 2012) and connected to future motivation in relationships (Salo et al., 2009). As a kind of scarce resource, knowledge can generate attraction in the context of innovation. The depth and the breadth of an organization’s activated knowledge determine its potential knowledge attraction to other network actors. A profound activated knowledge depth indicates that an organization has significant activated knowledge within a field, so it may have more attractiveness to other network actors that need the knowledge to achieve the technology-innovation goal. A wide activated knowledge breadth means that a wide range of fields are covered by an organization’s activated knowledge, meaning that there are more possibilities for it to attract other network actors. Knowledge attraction gives the other companies motivation to start a relationship with the owner and maintain it in the future. Therefore

#### Proposition 1

The more profound the focal organization’s activated knowledge depth is, the more potential knowledge attractiveness it has to other network actors.

#### Proposition 2

The wider the focal organization’s activated knowledge breadth is, the more potential knowledge attractiveness it has to other network actors.

2. At the dyadic level: studies in the field of psychology have found that as relative ability declines, the party that originally held more power will give up some power (Lindell and Campione-Barr, 2017). In other words, the shifting of capability will change the balance of inter-organizational power. Activated knowledge is defined in this paper as a result of capability and can generate and change relative power between two parties. Those with expertise can gain access to more alternatives; at the same time, this increases the certainty of access to alternative options, so they feel more empowered (Rlp et al., 2019), and they do have more power in negotiation and other aspects (Elfenbein, 2015; Wright et al., 2016; Schaerer et al., 2020). The emergence of greater psychological and behavioral power is closely related to attraction and dependence (Hald et al., 2009; Ramsay and Wagner, 2009). The larger the knowledge attractiveness an organization has for other network players, the more it is possible for the organization to be depended on and to acquire positive RKP. From potential knowledge attractiveness to effective RKP, however, there are two conditions: the other party must demand the activated knowledge that the focal actor has, and it must be difficult for it to acquire the knowledge from somewhere else. These conditions are indeed implied in AKCs at the dyadic level. Gaining complementary resources is an important inducement for organizations to enter into cooperation, as complementary resources will increase the interdependence between organizations (Pfeffer and Gerald, 1978). Thus, when an organization has a high level of activated knowledge that is complementary to the other party’s knowledge, this leads to the dependence of the latter on the former, which increases the possibility for an asymmetric dependence and consequent RKP to exist. However, if the focal organization’s activated knowledge can be easily substituted for by a third-network actor, the other party’s dependence will be distributed, which reduces the focal organization’s RKP. Therefore

#### Proposition 3

In a dyadic relation, the more the focal organization’s activated knowledge is complementary to the other party’s knowledge, the more RKP it has over the other party.

#### Proposition 4

In a dyadic relation, the more the focal organization’s activated knowledge can be substituted for by a third-network actor, the less RKP it has over the other party.

3. At the network level: power can be generated by social status. First, people with high social status enjoy respect and appreciation, resulting in others voluntarily giving them preferential treatment (Ball and Eckel, 1996). Second, people with high social status enjoy more social capital, e.g., in terms of being able to reach a wider range of negotiable objects. In order to obtain more social capital through them, other people will also give them power (Kim and Fragale, 2005). In an innovation network, companies with numerous power advantages have such a high social status and, thus, have absolute power. When an organization can acquire positive knowledge power from various dyadic relations with most network players, it will accumulate considerable NKP. This accumulating effect is ultimately demonstrated by the impacts of its AKCs on its NKP. The rarer an organization’s activated knowledge is in the whole network and the more important its knowledge is to the technology-innovation task, the more likely it is that the organization enjoys overall knowledge advantages over the other network actors. There is, therefore, a large probability of it acquiring positive RKPs and consequently considerable NKP and of occupying a central position with high social status in the knowledge-power network. Therefore,

#### Proposition 5

In the network, the more the focal organization’s activated knowledge rarity is, the more NKP it has.

#### Proposition 6

In the network, the more the focal organization’s activated knowledge centrality is, the more NKP it has.

**Figure 2:**
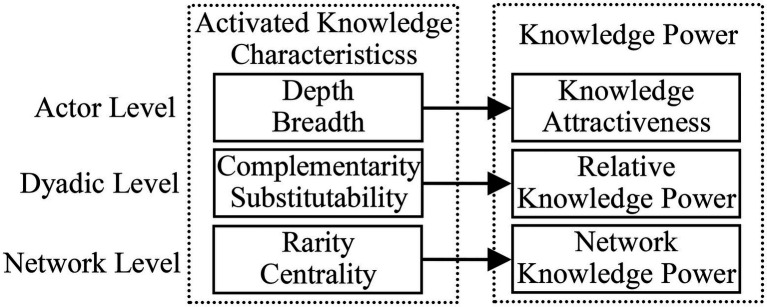
The concept model of AKCs’ impact on knowledge power.

The overall concept model is shown in Figure 2.

## Measurement Model

Based on the above analyses and taking the inter-organizational dependence equation proposed by Dastmalchian (1986) as groundwork, this section builds a multilevel measurement model for knowledge power and tests the above propositions by mathematical inference. The model incorporates the following assumptions:

Assumption 1: In an innovation network, inter-organizational collaborations are formed to accomplish a given technology-innovation task that requires a knowledge portfolio set as $K_{0}=\left[k_{01},k_{02},\ldots ,k_{0n}\right]$, with *n* for the breadth of the knowledge required and *k_0r_* for the depth of knowledge *r* needed to fulfill the task. For the sake of simplicity, the importance of different items of knowledge to the technology-innovation task is ignored by setting the weight of each item of knowledge’s importance as *1*.

Assumption 2: In the network, there are *m* organizations, and they know the other actors’ activated knowledge structure very well. That is because: (1) as defined above, activated knowledge refers to knowledge that is activated by organizational capabilities, embodied through technology-innovation activities or outcomes, and able to be sensed and identified by outsiders; (2) organizations in the same innovation network are usually located in one industry or homogeneous zones and interact frequently, which increases their number of mutual acquaintances; and (3) when an organization cannot achieve a technology-innovation goal by itself, it will be willing to let its potential partners know its knowledge advantages, with the aim of attracting them to cooperate. Thus, this assumption is reasonable.

### At the Actor Level

In an innovation network, power is derived from organizational knowledge, as mentioned above, and organizational activated knowledge is the precondition for knowledge power. Set $K_{i}=\left[k_{i1},k_{i2},\ldots ,k_{in}\right]$, with *k_ir_* for the depth of organization *i*’s knowledge *r*. *K_i_* reflects organization *i*’s activated knowledge depth and breadth in terms of the technology-innovation task, i.e., its activated knowledge.

Set


(1) $$Y_{i}=\left[y_{i1},y_{i2},\ldots y_{in}\right],\left(y_{ir}=\left\{\frac{1,\;\;k_{ir}\geq k_{0r}}{0,\;\;k_{ir}<k_{0r}}\right\},r\in \left[1,\;\;n\right]\right)$$

*y_ir_* indicates whether organization *i* is qualified in knowledge *r* according to *K_0_*, with *0* for no and *1* for yes. Thus, *Y_i_* represents the degree to which organization *i* can meet the technology-innovation goal. Consequently, $\left[1-y_{i1},1-y_{i2},\ldots ,1-y_{in}\right]$ reflects the set of knowledge that organization *i* needs to acquire from cooperation. The more chances *i* has to achieve the goal, the more attractive it is to other network actors for knowledge cooperation, and the more critical it is to the whole network. As *Y_i_* is directly impacted by *K_i_*, *i*’s potential knowledge attractiveness is in direct proportion to *i*’s activated knowledge depth and breadth. Proposition 1 and Proposition 2 are met.

### At the Dyadic Level

At this level, knowledge power is manifested as the RKP caused by asymmetric knowledge dependencies between organizations. Dastmalchian (1986) proposes the equation Dependence=Essentiality*1/Substitutability, with substitutability determined by the number of sources of resources available. The essentiality of organization *i*’s knowledge *r* to organization *j* relies on the following factors: if organization *i*’s activated knowledge *r* meets the technology-innovation requirement (*y_ir_*), if organization *j* needs to acquire knowledge *r* from outside (*1–y_jr_*), and the degree to which organization *j* depends on organization *i*’s activated knowledge *r* (*k_ir_ – k_ir_*). Suppose the dependence of organization *j* on organization *i* is *d_ji_*:


(2) $$d_{ji}=,\overset{n}{\underset{r}{\sum }}\left[y_{ir}*\left(1-y_{jr}\right)*\left(k_{ir}‐k_{jr}\right)*\frac{1}{\overset{m}{\underset{i}{\sum }}y_{ir}}\right],\left(r\in \left[1，n\right]\right)$$

Inter-organizational relative power is measured by the difference between their dependencies on each other (Pfeffer, 1981; Kumar et al., 1995). Let *p_ij_* be *i*’s RKP over *j*, and we get


(3) $$p_{ij}=d_{ji}–d_{ij}$$

By substituting Equation (2) into Equation (3), we get


(4) $$p_{ij}=,\overset{n}{\underset{r}{\sum }}\left[\left(k_{ir}-k_{jr}\right)*\left(y_{ir}+y_{jr}‐2y_{ir}y_{jr}\right)*\frac{1}{\overset{m}{\underset{i}{\sum }}y_{ir}}\right],\left(r\in \left[1，n\right]\right)$$

Set


(5) $$C_{ij}=\left[\begin{array}{l} \left(k_{i1}-k_{j1}\right)*\left(y_{i1}+y_{j1}-2y_{i1}y_{j1}\right),\left(k_{i2}-k_{j2}\right)*\\ \left(y_{i2}+y_{j2}-2y_{i2}y_{j2}\right),\ldots ,\left(k_{in}-k_{jn}\right)*\left(y_{in}+y_{jn}-2y_{in}y_{jn}\right) \end{array}\right]$$

*(y_ir_ +y_jr_ – 2y_ir_y_jr_)* is the residual part of the union of *i* and *j*’s activated knowledge sets after subtracting their intersection, i.e., the degree to which *i* and *j*’s activated knowledge are complementary in terms of knowledge breadth; *(k_ir_–k_jr_)* represents the degree to which *i*’s activated knowledge *r* is complementary to *j*’s in terms of knowledge depth. Therefore, *C_ij_* is indeed *i*’s activated knowledge complementarity to *j*’s.

Set


(6) $$NS=\left[ns_{1},ns_{2},\ldots ,ns_{n}\right],\left(\begin{array}{l} ns_{r}=\left\{\begin{array}{c} \frac{1}{\overset{m}{\underset{i}{\sum }}y_{ir}},\overset{m}{\underset{i}{\sum }}y_{ir}\neq 0\;\\ M,\overset{m}{\underset{i}{\sum }}y_{ir}=0 \end{array}\right.,\\ r\in \left[1，n\right],M\;is\;a\;non-zeroconstant \end{array}\right)$$

$\overset{m}{\underset{i}{\sum }}y_{ir}$is the total number of organizations that are qualified in knowledge *r* according to *K_0_*, and represents the substitutability of knowledge *r* (Dastmalchian, 1986). Further, *nsr*, as its reciprocal, refers to knowledge *r*’s non-substitutability. When $\overset{m}{\underset{i}{\sum }}y_{ir}=0$, the value of $\left(y_{ir}+y_{jr}‐2y_{ir}y_{jr}\right)$ must be zero, so the exact value of *M* has no impact on the result.

By substituting Equations (5)–Equations (6) into Equation (4), we get


(7) $$p_{ij}=C_{ij}{}^{T}NS$$

Therefore, organization *i*’s RKP over *j* is directly proportional to *i*’s activated knowledge complementarity to *j*’s, and is in inverse proportion to its activated knowledge substitutability (in direct proportion to its activated knowledge non-substitutability). Proposition 3 and Proposition 4 are met.

### At the Network Level

At this level, knowledge power is manifested as the accumulation of an organization’s RKPs in the knowledge-power network, i.e., its NKP. Let *p_i_* be organization *i*’s NKP, and we get


(8) $$p_{i}=\overset{m}{\underset{j}{\sum }}p_{ij}$$

By substituting Equation (4) into Equation (8), we get


(9) $$p_{i}=\overset{m}{\underset{j}{\sum }}p_{ij}=\overset{m}{\underset{j}{\sum }}\overset{n}{\underset{r}{\sum }}\left[\left(k_{ir}-k_{jr}\right)*\left(y_{ir}+y_{jr}-2y_{ir}y_{jr}\right)*us_{r}\right],\left(r\in \left[1,n\right]\right)$$

To simplify the mathematical inference and to highlight the major determining factors, this paper substitutes *(k_ir_–k_jr_)* for *(y_ir_–y_jr_)* in Equation (9) with the following justification: although NKP is derived from the accumulation of RKPs, as demonstrated in Equation (8), in reality, it is ultimately embodied by the organization’s position in the knowledge-power network. Indicators of network position such as centrality are often used to measure network power (Cook, 1977; Brass and Burkhardt, 1992; Ibarra, 1993; Mehra et al., 2006), and the nature of RKPs, as being positive or negative, has a much more significant influence on an organization’s network position than their magnitude. To a knowledge demander that has a certain degree of activated knowledge but that is inadequate to the technology-innovation goal, for example, although its existing knowledge may reduce its dependence on other qualified organizations, the fact cannot be changed that it still needs to depend on others for the technology-innovation task. Therefore, it will initiate collaborations with knowledge suppliers and try to maintain cooperative relations with them. This will increase the knowledge suppliers’ indegree centrality, which is a major index of a network actor’s power. The value of *(y_ir_–y_jr_)* reveals the nature of *i*’s RKP over *j* on knowledge *r*, as being at a power-advantageous (positive value), disadvantageous (negative value), or balanced (zero) position, and determines whether an organization is a knowledge demander or a knowledge supplier. So, although *(k_ir_–k_jr_)* can imply more about the NKP’s quantity, most of the information that it contains can be explained by *(y_ir_–y_jr_)*. Therefore, by substituting *(k_ir_–k_jr_)* for *(y_ir_–y_jr_)* in Equation (9), we get


(10) $$\begin{array}{l} p_{i}=\overset{n}{\underset{r}{\sum }}p_{ir}=\overset{n}{\underset{r}{\sum }}\left(\frac{m*y_{ir}}{\overset{m}{\underset{j}{\sum }}y_{jr}}-1\right)\\ \;\;\;\;\;\;\;\;\;\;\;\;\;\;\;\;\;\;=\overset{n}{\underset{r}{\sum }}\left(y_{ir}*\frac{m}{\overset{m}{\underset{j}{\sum }}y_{jr}}\right)-n,\left(r\in \left[1,n\right]\right) \end{array}$$

Set


(11) $$S=\left[s_{1},s_{2},\ldots ,s_{n}\right],\left(\begin{array}{l} s_{r}=\left\{\begin{array}{c} \frac{m}{\overset{m}{\underset{i}{\sum }}y_{ir}}=\frac{1}{\frac{\overset{m}{\underset{j}{\sum }}y_{jr}}{m}},\overset{m}{\underset{i}{\sum }}y_{ir}\neq 0\;\\ N,\;\;\;\overset{m}{\underset{i}{\sum }}y_{ir}=0 \end{array}\right.,\\ r\in \left[1,n\right]\;and\;N\;is\;a\;non-zeroconstant \end{array}\right)$$

The fraction $\frac{\overset{m}{\underset{j}{\sum }}y_{jr}}{m}$, with the denominator being the total number of organizations in the network and the numerator being the total number of qualified organizations in knowledge *r*, represents the density of the organizations with qualified knowledge *r* in the network. Further, *S*, as its reciprocal, reflects the rarity of knowledge *r* in the network.

By substituting Equation (11) into Equation (10), we get


(12) $$p_{i}=Y_{i}^{T}S‐n\;\left(r\in \left[1,n\right]\right)$$

As *n* is a constant, the value of *p_i_* is determined by *Y_i_* and *S*. *Y_i_* represents whether an organization *i*’s various types of knowledge are qualified for the technology-innovation task and measures its knowledge centrality in the network. *S* represents knowledge rarity, as analyzed above. Therefore, organization *i*’s NKP is determined by *i*’s activated knowledge centrality as well as its activated knowledge rarity. Proposition 5 and Proposition 6 are met.

## Conclusion

### Findings

The KBV is a major perspective for studying innovation networks, as it captures nicely the feature of technology-innovation networks that knowledge is the key resource. Relying exclusively on the KBV, however, overlooks both the multilevel structure of the network and network actors’ active role and can hardly reveal how the network, as a spontaneous system, evolves into a macro structure through interactions among the actors through micro activities. To provide a solution to these problems, the concept of knowledge power is introduced here, based on related theories. This paper suggests that knowledge power reflects the nature of inter-organizational relations in networks, fits their multilevel character, and matches the latest findings that inter-organizational power relations are asymmetric and that innovation networks are hierarchical. Studies of knowledge power can provide a novel clue for research on the static structure as well as the dynamic evolution of innovation networks. Grounded in the above reasons, this paper initiates some basic analyses of knowledge power as follows:

1. Knowledge power’s origins in knowledge and capability are analyzed, and by combining the multilevel feature of the innovation network, knowledge power’s formation path is described;
2. The concept of activated knowledge, which is thought to be the major determinant of knowledge power, is proposed; a multilevel framework for activated knowledge’s characteristics is built; and their impacts on knowledge power are analyzed; and
3. Measurement equations for knowledge power at different levels are deduced by taking a well-accepted dependence equation as the groundwork; the above propositions are tested; and a multilevel measurement model for knowledge power is established.

**Figure 3:**
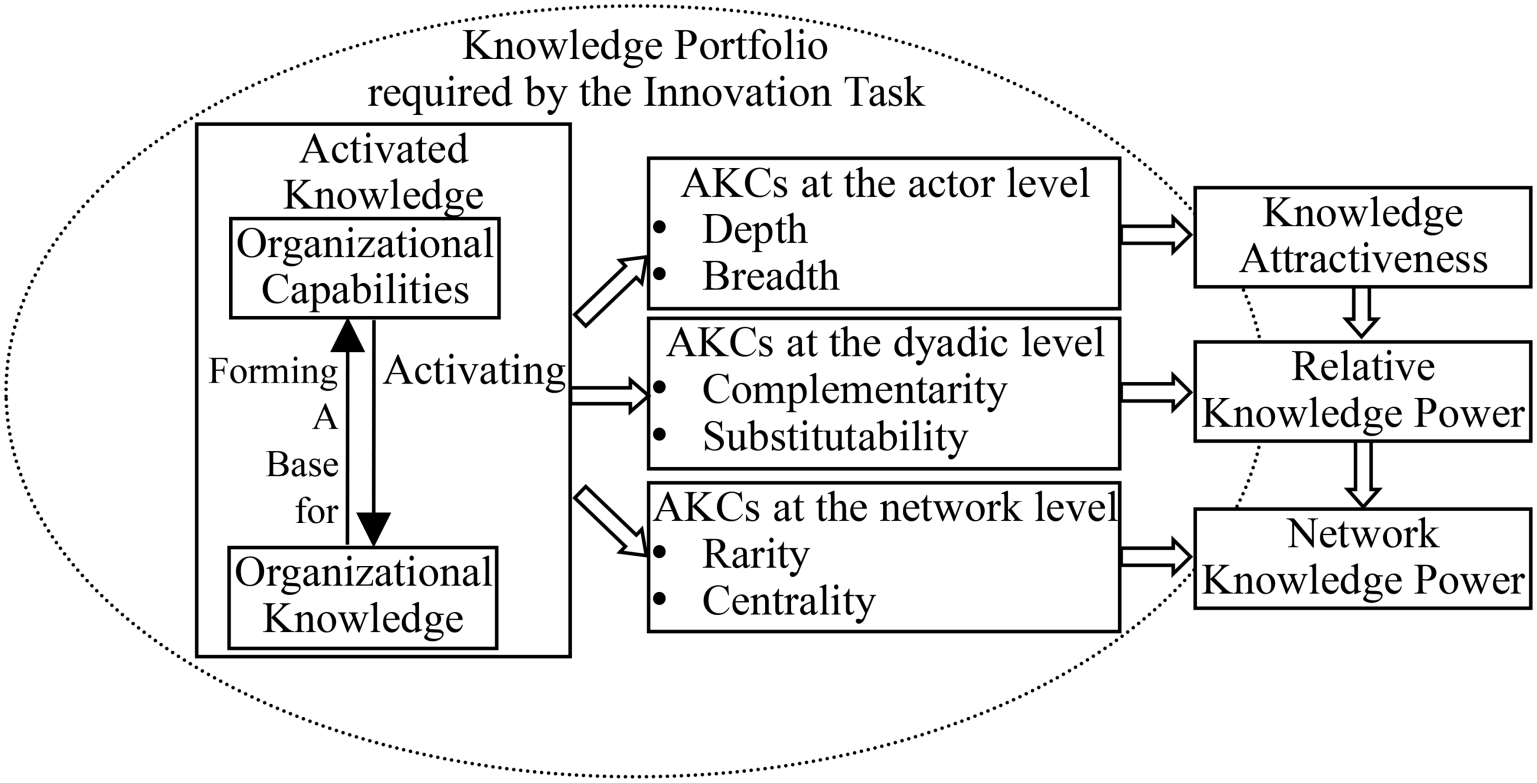
Knowledge power in the innovation network.

The major work of this paper is shown in Figure 3.

### Research Limitations/Implications

The concept of knowledge power inherits the well-accepted feature of technology-innovation networks that knowledge is the most important element and captures the newly revealed phenomenon that power relations in the network are asymmetric. This is a long-overlooked research topic in technology-innovation-network studies that has received little systemic investigation. This paper analyzes knowledge power’s formation path, proposes propositions for its determining factors, and builds a measurement model. However, as a fundamental analysis of an almost entirely new concept, this paper has some deficiencies; to address them, future research can be conducted in the following areas:

1. Empirical studies can be conducted to test AKCs’ impacts on knowledge power on multiple levels to validate the propositions proposed by this paper with practical data.
2. Based on the static measurement model of knowledge power, a dynamic model may be developed to simulate knowledge power’s formation and changes and to provide a groundwork for further studies of network evaluation.
3. Based on inter-organizational resource dependence theory, this paper combines the KBV and the dynamic capability theory to explore the formation mechanism from knowledge to power. However, the relational view derived from the sociology field is also an important perspective for studying inter-organizational relationships and proposes some important variables such as trust, reciprocity, communication, etc. Future research may explore the interactions between these variables and knowledge power to arrive at a better understanding of inter-organizational relationships in innovation networks.

### Practical Implications

The members of an open innovation network are loosely coupled, and the interactions among the members form a dynamic network. Such interactions are greatly impacted by members’ visibility and authority in the network, the availability of network resources, and the dynamics of the overall goals of the network. Power is a key factor interwoven with these issues, especially in innovation networks, which usually have some core actors. Gaining a better understanding of the structure and determinants of knowledge power is crucial to the success of network members. The analysis and outcomes of this paper may:

1. Inspire a power-disadvantaged network actor to actively improve its influence and importance in the network and to change its position, both in dyadic rations and in the overall network, by improving its own knowledge and capabilities;
2. Remind a power-advantaged network actor to apply its RKP and NKP reasonably to spur a next-stage technology-innovation task in favor of its own AKCs, so as to anchor its power-advantageous position; and
3. Make it easier to identify network actors’ power by distinguishing their AKCs, as proposed in this paper, and to maintain an overall coordinated and sustainable network development by emphasizing the active roles of network actors, especially those with a great degree of power.

## Author Contributions

SC: ideas, formulation of the research goals, design of methodology, creation of models, verification of the research outputs, investigation of the outcomes, application of mathematical techniques to analyse, creations of the original draft preparation, management for the research activity planning, execution and revision, and acquisition of the financial support for the projects. ZF: coordination for the revision process and review and editing the revised manuscript before it were formally edited by the professional proofreading servicer. ZP: search, analysis, and interpretation of related and up-to-date references and pre-formatting the references list before the manuscript was formally edited by the professional proofreading servicer. SQ: search, analysis, and interpretation of related and up-to-date references and pre-formatting the references list before the manuscript was formally edited by the professional proofreading servicer.

## Funding

This research was funded by the Key Research Institute of Philosophy and Social Science of the Education Department of Shaanxi Provincial Government (No. 18JZ010), Research Project on Major Theoretical and Practical Problems of Philosophy and Social Sciences in Shaanxi Province (No. 2021Nd0029), Xi’an Social Science Planning Fund Project (No. Gl51), and Science and Technology Innovation Team of Innovative Talent Promotion Plan in Shaanxi Province (No. 2021TD-35).

## Conflict of Interest

The authors declare that the research was conducted in the absence of any commercial or financial relationships that could be construed as a potential conflict of interest.

## Publisher’s Note

All claims expressed in this article are solely those of the authors and do not necessarily represent those of their affiliated organizations, or those of the publisher, the editors and the reviewers. Any product that may be evaluated in this article, or claim that may be made by its manufacturer, is not guaranteed or endorsed by the publisher.

## Footnotes

^1^The term “power network” was coined by Emerson (1962) and refers to two or more connected power-dependence relations.
